# Optimization of Conventional Extraction Parameters for Recovering Phenolic Compounds from Potato (*Solanum tuberosum* L.) Peels and Their Application as an Antioxidant in Yogurt Formulation

**DOI:** 10.3390/antiox11071401

**Published:** 2022-07-19

**Authors:** Fatiha Brahmi, Inmaculada Mateos-Aparicio, Alejandra Garcia-Alonso, Nadjet Abaci, Salima Saoudi, Leila Smail-Benazzouz, Hayate Guemghar-Haddadi, Khodir Madani, Lila Boulekbache-Makhlouf

**Affiliations:** 1 Laboratory of Biomathematics, Biochemistry, Biophysics and Scientometry, Faculty of Natural and Life Sciences, University of Bejaia, Bejaia 06000, Algeria; abbacinadjet@gmail.com (N.A.); sisasirina56@gmail.com (S.S.); leila.benazzouz@univ-bejaia.dz (L.S.-B.); hayate.haddadi@univ-bejaia.dz (H.G.-H.); khodir.madani@univ-bejaia.dz (K.M.); lila.makhlouf@univ-bejaia.dz (L.B.-M.); 2 Department of Nutrition and Food Science, Universidad Complutense de Madrid, 28040 Madrid, Spain; alejandra.garcia.a@ucm.es; 3 Agri-Food Technologies Research Center, Targua Ouzemmour Rouad, Bejaia 06000, Algeria

**Keywords:** optimization, extraction, potato peels, polyphenols, antioxidant activity, yogurt

## Abstract

The aim of this work was to optimize the conventional parameters for the extraction of phenolic compounds from potato (*Solanum tuberosum* L.) peels (PP). A central composite design (CCD) was used to establish the impacts of ethanol concentration (%), extraction time (min), and liquid/solid ratio (mL/g). The optimal experimental conditions that maximized extraction were ethanol at a concentration of 80% (*v/v*) for a time of 150 min with a ratio of 1 g/30 mL. Under optimal conditions, the total phenolic content (TPC) and the total flavonoid content (TFC) were 204.41 ± 8.64 mg GAE/100 g DW and 21.47 ± 0.76 mg QE/100 g DW, respectively. The PP extract had a potent antioxidant capacity tested by phosphomolybdate and DPPH assays with IC_50_ of 10.65 ± 0.21 and 179.75 ± 3.18 µg/mL, respectively. Furthermore, by fortifying yogurt with PP as a natural ingredient, an improvement ofits physical, nutritional, antioxidant, and sensorial qualities was attempted in this study. The yogurts formulated with PP revealed significantly higher (*p* ≤ 0.05) TPC, TFC, and antioxidant capacity in comparison with the control sample. In addition, the sensory evaluation showed that the yogurts enriched with PP were preferred over the control yogurt. The results indicate that PP can be considered an interesting byproduct since it can improve the nutritional, bioactive, and sensorial profile of yogurt, highlighting that PP, due to its high phenol content, can substantially improve the antioxidant effect of the new formulated yogurt.

## 1. Introduction

Potato (*Solanum tuberosum* L.) is the world’s first non-grain food commodity [1] and is one of the most extensively edible crops in the world. Currently, methods of consumption of potato are shifting from fresh to processed, such as chipped potatoes. This has engendered environmental concerns related to the wastes generated by such manufacturing procedures [2]. Among these wastes, potato peel is the most important one, but it is also a high resource of bioactive substances with antioxidant activity, i.e., phenolic compounds [3], and thus should be considered a byproduct to reuse following the principles of a circular economy.

The extracted polyphenols from vegetables are acknowledged as the most auspicious category of substances able to maintain the nutritional and sensorial product characteristics. Moreover, these compounds can protect human cells from disproportionate oxidation and damage caused by freeradicals. Indeed, a negative correlativity among the consumption of dietary antioxidant components such as phenolics and pathologies has been found [2,4,5]. Thus, the search for sources of these compounds and methodologies to obtain them is of key interest to the food, pharmaceutical, and cosmetic industries.

A substantial feature of the extraction of phenolics from plants is the choice of the relevant extraction parameters. It is important to study and evaluate the parameters due to the vegetable’s matrix diversity and the different and variable phenolic structures, and thus, it is not recommended to adopt the same extraction parameters for different categories of plants [6].

The extraction technique is a restrictive procedure, as the initial phase affects the separation of phenolic compounds from vegetables. The extraction by maceration, a conventional method, remains the main process to extract phenolics from different plants because more recent technologies often have an elevated investment cost. It is a simple technique using non-complicated equipment, and one that does not require a skilled operator. In addition, this conventional method is an energy-saving process and is ideal for some molecules that are very poorly soluble in solvent and need only extended contact with it [7]. Otherwise, each method has its own advantages and disadvantages; however, the main objective of the selected technique is the accomplishment of the complete extraction of phenolic compounds and the prevention of their chemical alteration.

Solid–liquid extraction of phenolic compounds involves the choice of adequate solvents. Since polyphenols are made up of several hydroxyl groups, polar solvents such as ethanol or aqueous ethanol are the best choice for their extraction [8].

Most literature addresses the optimization of phenolic extraction parameters from potato peel using ultrasound-assisted extraction [2,3,9,10] or pulsed electric fields-assisted extraction [4]. Lakka et al. [11] carried out a maceration method, but used the green solvent hydroxypropyl β-cyclodextrin. However, to the authors’ knowledge, no work has addressed the optimization of conventional extraction conditions of phenolic compounds from Algerian potato peels, taking into account, as discussed, the fact that phenolic compounds have a favorable impact on protecting against the oxidation of foods, such as dairy products, increasing their shelf life, and also as antioxidants for the human body [12,13].

Yogurt is one of the main dairy products developed and consumed worldwide [14]. This dairy product is renowned as a prevalent functional food with interesting nutritional, sensorial, and healthy attributes that seem be related to strengthening the immune system due to its chemical composition, namely, probiotic bacteria, proteins, lactose, minerals, and water-soluble vitamins. However, it lacks phenolic compounds, although other antioxidant substances such as peptides from milk protein are present. In any case, consumer demand for functional and healthy foods has increased enormously [13,15], and the enrichment of yogurt with natural bioactive compounds from various vegetables has attracted the interest of scientists and manufacturers; as a result, innovative functional yogurts have been developed that possess enhanced health-promoting properties. Anuyahong et al. [16] demonstrated that acute consumption of yogurt enriched with anthocyanins from rice berry decreased postprandial glucose content and improved plasma antioxidant capacity in healthy volunteers. Purple sweet potato used to fortify yogurt has improved its functional values [17], and adding 15% pumpkin pulps enhanced some chemical, physicochemical, and sensory characteristics, as well as the antioxidant capacity of stirred yogurt [18]. Similarly, yogurt enriched with hazelnut skin showed enhanced bacteria viability, water holding capacity, and antioxidant capacity [19].

Interestingly, a phenolic-rich solution resulting from wine was used to prepare a yogurt, which was characterized by attractive organoleptic properties and appreciable antioxidant activity [20]. Yogurt produced by the incorporation of the cryo-concentrated strawberry pulp has three-fold more anthocyanins and antioxidant effects [21]. A new bioactive yogurt enriched with olive fruit polyphenols was also found acceptable by customers and had a protective impact towards undesired pH drop throughout yogurt preservation [22]. In addition, the antioxidant capacity of the yogurt beverages fortified by aqueous extracts of dried berry fruits and skins as origins of phenolics was noticed [23], and common purslane extract is considered a good source of functional compounds for yogurt fortification [24].

Several other natural products have been added advantageously in the enrichment of yogurt, including apple peel polyphenol [25], argel (*Solenostemma argel* Hayne) leaf extract [13], *Spirulina platensis* [15], grape (*Vitis labrusca* L.) pomace [26], juices from grapes and berries [27], red ginseng extract [28], psyllium (*Plantago ovate*) husk [29], *Pleurotus ostreatus* aqueous extract [30], apple pomace [31] and apple peels, and grape seed powder [32].

Therefore, the integration of phenol-rich natural products into yogurt improves its bioactive profile and increases the antioxidant capacity of yogurt compared to plain yogurt [13]. However, to the authors’ knowledge, the application of potato peel waste in dairy products has not yet been achieved.

The present study aimed to select the most adequate conventional extraction parameters (ethanol concentration, temperature, and the liquid/solid ratio) of total phenolic compounds from the peels of potato growing in Algeria. Then, the dried potato peels were used as a polyphenol source to formulate a new yogurt. The physicochemical and sensorial properties, total phenolic content (TPC), total flavonoid content (TFC), and the antioxidant capacity were assessed and compared between the yogurts prepared with or without the dried potato peel byproduct.

## 2. Materials and Methods

### 2.1. Materials

The potatoes were purchased from the market in Bejaia (Algeria), and then were well cleaned, wiped, and peeled. The potato peels (PP), with a moisture content of 87%, were dried in a convection oven at 40 °C for 72 h. Then, they were ground into powder (IKA A11 Analysis Mill, Staufen, Germany), which was sieved to obtain particles with a diameter ≤ 500 µm. This powder was stored at 20 °C in opaque glass bottles until used.

All of the chemicals used were of reagent grade, purchased from Biochem Chemopharma (Montreal, Quebec) or Sigma-Aldrich Co. (St. Louis, MO, USA), and employed without additional handlings.

### 2.2. Extraction

The PP powder (0.5 g) was extracted with 10 mL of ethanol (20, 40, 60, 80, and 100%, *v/v*) in a glass cylinder, and then shaken by magnetic stirring. The extraction times were 30, 60, 90, 120, 150, and 180 min. The mixture was centrifuged at 1765 g for 10 min after extraction and the extract was then retrieved.

### 2.3. Quantification of Total Phenolic (TPC) and Total Flavonoid (TFC) Contents

The TPC in the PP extracts were measured following the Folin–Ciocalteu method reported previously [32]. The PP extract (100 µL) was mixed with 500 µL of Folin–Ciocalteu reagent, followed by adding 1500 µL of sodium bicarbonate solution (20%, *w/v*). The mixture was vortexed and incubated at room temperature for 60 min. The absorbances were determined at 760 nm (UV-Vis Spectrophotometer, spectro scan 50 Shimadzu, Kyoto, Japan) and gallic acid (GA) was used as standard. The TPC was calculated as milligrams of gallic acid equivalents (GAE) per 100 g dry weight of PP powders (mg GAE/100 g DW).

The TFC in the PP was quantified after mixing extracts (1 mL) with 1 mL of aluminum chloride (2%, *w/v*). After incubation at room temperature for 15 min, the absorbance was determined at 430 nm. The TFC was expressed as milligram quercetin equivalent (QE) per 100 g DW of PP (mg QE/100 g DW) [32].

### 2.4. Determination of Antioxidant Capacity

The antioxidant capacity of the PP extract was performedby the phosphomolybdate ion reduction and the DPPH radical scavenging assays.

In the total antioxidant capacity measured by the phosphomolybdate method, Mo (VI) is reduced to Mo (V) in the presence of antioxidants and forms a green-colored phosphomolybdenum V complex, which has a maximum absorbance at 695 nm. This assay was assessed by mixing 0.2 mL of PP extract with 1 mL reagent solution prepared with 0.6 M sulfuric acid, 28 mM sodium phosphate, and 4 mM ammonium molybdate. The samples were incubated in a water bath for 90 min, and after that, the absorbance was determined at 695 nm and the data were expressed as IC_50_ (μg/mL) [32].

The free radical-scavenging activity (RSA) of the PP extract on 2,2-diphenyl-1-picrylhydrazyl (DPPH^•^) radicals was measured as previously described [32]. An aliquot (50 µL) of PP extract was added to DPPH (150 µL, 10^−1^ mM). Decolorization was evaluated by measuring the absorbance (Abs) at 517 nm after 30 min of reaction. The RSA was determined as percentage (%) inhibition using Equation (1):

DPPH^•^ RSA (%) = [(Abs_control_ − Abs_sample_)/Abs_control_] × 100 (1)

The activity is expressed as the concentration of the compound that gives 50% scavenging of DPPH^•^ radicals defined as IC_50_ (µg/mL) values. These values were calculated by plotting inhibition percentages against extract concentrations.

### 2.5. Formulation and Characterization of PP Yogurt

Yogurt was prepared with fresh cow’s milk containing 3% fat, which was heated at 90 °C for 10 min and then cooled to 45 °C. At that point, the starter culture, a combination of *Streptococcus thermophilus* and *Lactobacillus delbrueckii* subsp. *bulgaricus*, was added. After the milk was inoculated with the starter culture, dried PP in the form of powder or small pieces of 5 mm^2^ (0.8 g) were added to 100 g milk, and it was then stirred gently. The mixture was fermented at 45 °C until a final pH of 4.5 and cooled at room temperature. Then, all yogurt samples were stored at 4 °C until analyzed. Control yogurt samples were prepared by inoculation of homogenized milk.

The characterization of the PP yogurt consisted of the determination of moisture, ash, total solids, pH, acidity, TPC, TFC, antioxidant activity, and sensorial analysis.

Moisture was determined by drying samples at 105 °C until constant weight using the oven dry method as the reference method. Ash was evaluated using a furnace ashing technique, and total solid amounts were assessed using the dry oven method. pH was determined with a pH meter and total titratable acidity was determined by titration with standard 0.1 N NaOH until pH 8.1 was reached, and is expressed as g of lactic acid/100 mL milk. Phenolic compounds were extracted from the yogurt samples (20 g) with 30 mL of acidified water (0.1% HCI) at 4 °C overnight. The solution was then filtered through Whatman No.1 filter paper under vacuum. The TPC, TFC, and antioxidant capacity were determined with the same procedures described in Section 2.3 and Section 2.4.

The sensorial evaluation of the yogurt samples fortified with potato powder or pieces was carried out with the assistance of 10 trained panelists from the Department of Food Sciences (Bejaia University, Bejaia, Algeria), who were well skilled and familiar with yogurt: five males and five females (aged between 30 and 55). The sensorial properties of the yogurt were evaluated according to eight different categories, namely, color, smell, aroma, sweetness, acidity, texture, consistency, and general quality. Evaluation was scored on a 5-point hedonic scale (1: dislike extremely to 5: like extremely) for smell, aroma, color, sweetness, consistency, acidity, and texture. The overall quality of the samples was evaluated on a 9-point scale.

### 2.6. Experimental Design and Statistical Analysis

The extraction of TPC as a function of ethanol concentration (*E*), processing time (*T*), and liquid/solid ratio (*R*) was studied using a rotatable second-order design with six replicates in the centre of the experimental domain. The conditions of the independent variables studied were: *E* in the range 20–100%, *T* in the range 30–180 min, and *R* in therange 10–60 mL/g.

In this section, the experiment was divided into two parts. The initial part included the determination of the lower, middle, and upper levels of the three design variables employed in the RSM. These levels of independent variables were selected based on the values obtained in the preliminary experiments [32]. Hence, the first step of the preliminary experiment was to select the appropriate conditions for the extraction of PP phenolics. An early step was to assess the optimum ethanol concentration. The experiments were conducted under the chosen conditions for polyphenol extraction and relied upon previous study data [32].

The effect of ethanol concentration on the extraction was studied over a period of 60 min, with a 1/20 solid–solvent ratio and 500 µm particle size that was fixed over all of the experiments. The ethanol concentrations used were 20, 40, 60, 80, and 100%, *v/v*. In the next set of experiments, the impact of each variable on the extraction process was investigated and the results are shown in the preliminary screening. Each factor in all levels was separately tested in combination with the other studied variables.

To establish the appropriate extraction time of the PP phenolics, six levels, 30, 60, 90, 120, 150, and 180 min, were used. Thereafter, six levels of the solid–solvent ratio (1/10, 1/20, 1/30, 1/40, 1/50, and 1/60 g/mL) were evaluated to choose the appropriate ratio for extraction (Table 1).

The choice of the process levels of each variable that had a significant effect on the extraction of total phenolics (TP) was carried out. Selected levels with the highest yields of TP were exposed to further factorial designs.

In the factorial design (central composite design), the three independent variables: ethanol concentration, time, and solid–solvent ratio, each at two levels, were screened forming the 20 full factorial design (Table 2) to investigate their effects and choose the optimum values on the total phenolic content (dependent variable).

Each factor was tested at the two most promising levels using the upper and lower limits chosen based on the preliminary screening. The three factors and the lower, middle, and upper design points for RSM in coded and natural/ uncoded values are shown in Table 1.

In the second part, in RSM, natural variables were transformed into coded variables that were defined as dimensionless with a mean of zero and the same spread or standard deviation [33].

Both expressions of the independent variables, codified, and natural values, in each experimental run are summarized in Table 2.

Orthogonal leastsquares calculations on factorial design data were used to obtain, by means of orthogonal leastsquares calculation, empirical equations describing TPC (*Y*) related to *T*, *E*,and *R* effects. The general form of the polynomial Equation (2) is:

(2) $$Y=\beta_{0}+\sum_{i=0}^{k}\beta_{i}X_{i}+\sum_{i=1}^{k}\beta_{ii}X^{2}{}_{i}+\sum_{i>1}^{k}\beta_{ij}X_{i}X_{j}+E$$

where *Y* represents the TPC response to be modeled; *β*_0_ is the constant coefficient;*β_i_* is the coefficient of linear effect; *β_ij_* is the coefficient of interaction effect; *β_ii_* is the coefficient of squared effect; *k* is the number of variables; and *X_i_* and *X_j_* define the independent variables (*E*, *T*, and *R*). The statistical significance of the coefficients was verified by means of the Student’s t-test (α = 0.05), goodness-of-fit was established as the adjusted determination coefficient (R^2^_adj_), and the model consistency by the Fisher F test (α = 0.05).

The analysis of variance was conducted using STATISTICA, Version 10.0. All results are presented as the mean of triplicate measurements. The level of significance was set at *p* < 0.05.

The experimental design, model fitting, and processing of the data obtained from the implementation of the response surface methodology were carried out with JMP software (version 7.0.1, SAS Institute Inc., Cary, NC, USA). The treatment of the sensorial analysis data was determined by XL STAT (version 9.0, Addinsoft, Paris, France).

## 3. Results and Discussion

### 3.1. Selection of Ethanol Concentration, Time, and Liquid-to-Solid Ratio of Phenolics Extraction from PP

Initial assays were conducted to estimate the necessary ethanol concentration, time, and liquid-to-solid ratio for the recovery of the phenolic compounds from the PP by maceration.

**Figure 1:**
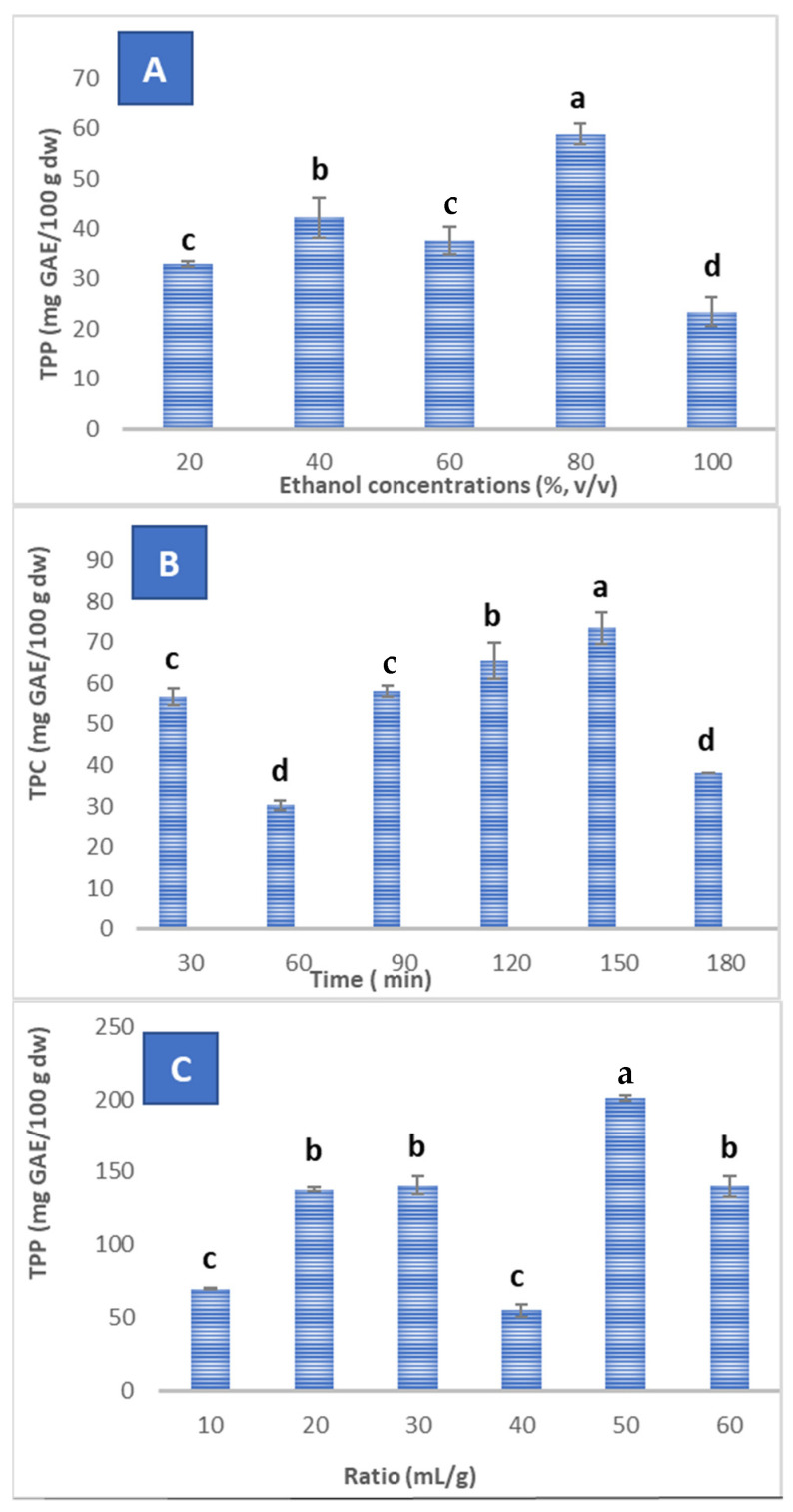
Influence of the ethanol concentration (**A**), time (**B**), and liquid/solid ratio (**C**) on the content of total phenolics in potato peel extracts. The mean of TPC assigned with different letters indicates significant differences (*p* ≤ 0.05); those followed by the same letter are not significantly different.

Different extracting solvents were employed for the TPC recovery from the PP, and ethanol was among the best ones since it possesses limited constraints for food utilization [8]. Therefore, ethanol–water was used for the extractions in the proportions 20, 40, 60, 80, and 100%, *v/v*. The results revealed that the TPC was dependent on the ethanol concentration, and it increased gradually with the increase of ethanol proportion from 20% (33.00 ± 0.56 mg GAE/100 g DW) to 80% (58.91 ± 2.14 mg GAE/100 g DW), where it reached its maximum and decreased thereafter (Figure 1A). So, ethanol at 80% (*v/v*) was the best concentration chosen for the phenolics extraction from the PP. 

For the extraction of phenolic compounds, alcoholic solvents are often used; however, they are not particularly specific, and a mixture of alcohol and water has been shown to be more effective. The addition of water to ethanol commonly generates a more polar media that promotes the extraction of phenolics, which are more soluble in polar solvents [34]. Water plays a substantial role in the swelling of vegetable matter, while ethanol is responsible for disrupting the bonding between the solutes and plant matrix; hence, it facilitates better mass transfer of the constituents. Thus, the mixture of water and ethanol as a solvent agent demonstrates a synergistic effect, which enables phenolic extraction [35].

Solvents with considerable polarity improve the extraction of phenolics. The use of the ethanol–water mixture is advisable in many studies compared to pure solvent [36].

Moreover, previous studies have shown that the use of very pure organic solvents can lead to the dehydration and collapse of plant cells, as well as the denaturation of cell wall proteins, thus making the extraction of phenolic compounds difficult [37].

Regarding time, the maximum TPC (73.50 ± 3.82 mg GAE/100 g DW) was obtained at 150 min, while the extracts obtained after 30, 60, 90, 120, and 180 min gave lower (*p* < 0.05) values (Figure 1B). Previous studies about the optimal extraction time are quite contradictory: some authors recommend short extraction times of 5 to 30 min [38,39], while others demonstrate the need for longer contact times of 1 to 24 h [40]. Nevertheless, too long a time, beyond 24 h, increases the possibility of phenolic oxidation and would consequently reduce the extraction yield [41].

The diffusion of the sample constituents such as phenolic compounds and mass transfer ratios are improved by longer extraction times, which allow a close and efficient connection between the solvent and the matrix. Extraction times up to 150 min are related to the extraction of phenolic compounds from different matrices [36]. After a certain time, the phenolic extraction yield decreases markedly. This can be explained by the degradation of some active compounds during the prolonged extraction time [33] and the oxidized compounds generated can turn into insoluble constituents [32].

The third parameter evaluated was the liquid-to-solid ratio. The TPC was conditioned by the liquid-to-solid ratio, being the best yield 1: 50 with a TPC of 201.25 ± 1.77 mg GAE/100 g DW, and the lowest one was obtained using a ratio of 1:40 (55.00 ± 1.24 mg GAE/100 g DW) (Figure 1C). The variation in the ratio influences the yield of total polyphenol extraction. The contents increased with increasing volume of solvent, as shown by Wang et al. [3], who found that the extraction yield increases with the increase in the liquid-to-solid ratio, with 60 mL of solvent being the necessary volume to extract the maximum phenolic compounds from the PP. The amount transferred from solid to liquid is conditioned by the concentration, so a rise in the liquid volume facilitates phenolics extraction. In fact, the TPC from the PP had a powerful correlation with the solvent-to-solid ratio [3].

A high solvent/solid ratio is often recommended for a complete extraction of all substances in a sample [36]. In fact, a high solid/solvent ratio leads to a decrease in the use of plant material and reduces extraction costs [35]. This high solvent/solid ratio is related to the principles of mass transfer, and thus, induces an increase in the diffusion rate. On the other hand, when the extractions are completed and equilibrium is reached, the solvent/solid ratio has no significant effect on the diffusivity and diffusion rate [32].

Other factors such as the extraction conditions, mainly solvent composition andtemperature, which affect solubility and solute–solvent interactions, can modify phenolic yields as well as the equilibrium constant [42].

### 3.2. Optimization of TPC Extraction

The associated impact of ethanol concentration (*E*), time (*T*), and liquid/solid ratio (*R*) of extraction was assessed using surface response methodology.

The TPC in the PP varied from 12.27 to 217.56 mg GAE/100 g DW, which confirms the influence of the parameters (*E*, *T*, and *R*) on the TP rate (Table 2). Kumari et al. [10] found that the TPC fluctuated from 217 to 328 mg GAE/100 g db and Amado et al. [43] from 34 to 113 mg/100 g. Additionally, according to Paleologou et al. [9], the TPC varied from 140 to 905 mg caffeic acid equivalents per 100 g DW.

The data from the TPC were transformed into a second-order polynomial equation as a function of three independent variables (*E*, *T,* and *R*), as follows:

*Y* = 42.57 + 21.98*E* + 38.81*T* + 13.23*R* + 17.02*ET* + 15.04*ER* + 8.16*TR* − 16.08*E*^2^ + 23.79*T*_2_^2^ + 42.99*R*_3_^2^ (3)

Equation (3) represented more than 98% of the response variation, signaling that these experimental results are in favor with the responses predicted and the lack of fit test was not significant (Table 3).

This model results a suitable predictor for the extraction of polyphenols from PP considering ethanol concentration (*E*), time (*T*) of extraction, and S/L ratio (*R*).

The regression coefficients, the ANOVA, and the coefficients of multiple determination (R^2^) for the TPC are given in Table 2. The results of the multivariate analysis revealed that the statistical significance of the coefficients was response-dependent and all parameters were significant (*p* < 0.05) for TPC recovery.

The response surfaces show the complex relationship between ethanol concentration, extraction time, and S/L on the TPC of the PP extracts. Indeed, although all of the parameters considered possessed a statistically significant impact on TPC (*p* ≤ 0.05), the time and ethanol concentrations were the variables that mainly affected the observed response. It is well known that a prolonged extraction time increases the extraction efficiency of TPC. However, when the threshold is reached, there is a reduction over time. A decrease in TPC was also recorded in the initial assays of this study, even when the extraction time was prolonged for more than 150 min. The decrease in TPC extraction after the threshold time may be the result of the degradation of phenolic compounds, as discussed above.

**Figure 2:**
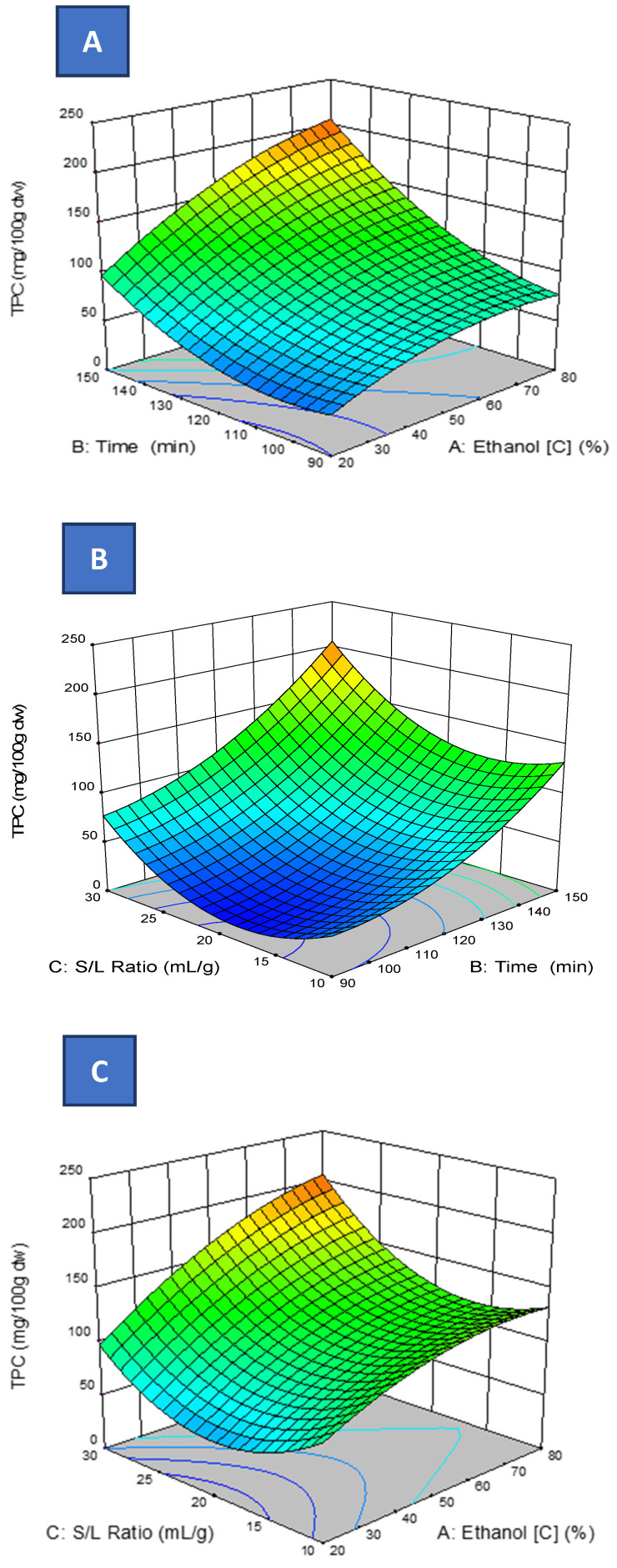
Response surface plot of the effect of ethanol concentration (%, *v/v*) and extraction time (min) (**A**), ethanol concentration (%, *v/v*) and liquid/solid ratio (mL/g) (**B**) and extraction time (min) and liquid/solid ratio (mL/g) (**C**) on the TPC of the PP extract.

Regarding the solvent proportion, the ethanol concentration impacts the polarity of the solvent combination, hence allowing the extraction of phenolics with a large range of polarities [4]. There were two different zones: a rising zone until almost 80% of ethanol, continued by a decline in the TPC yield, where a negative quadratic impact was noticed (*p* < 0.05) (Table 2 and Figure 2).

The interaction effect between the ethanol concentration and time (*E* × *T)* (Figure 2A) revealed that a low ethanol concentration and a short extraction time results in smaller (*p* < 0.05) TPC, but a positive impact on TPC was noticed using a high ethanol concentration for a longer time.

For the interaction *E* × *R* effect (Figure 2B), it can be outlined that low ethanol proportion and low S/L ratio restricts the TPC. This result is consistent with previous studies on the interaction impact of solvent concentration and L/S ratio on the extraction of TP from PP [4,9].

The augmentation in extraction yield using an elevated L/S ratio is perfectly described not only for phenolic extraction, but also for the recovering of other compounds [40]. The rise in L/S ratio leads to a high-intensity gradient between the solid and the adjacent liquid, and hence intensifies the distribution of solutes from the solid into the solution [3]; the principle of this phenomenon was described previously.

The interaction between time and S/L ratio (*T* × *R*) (Figure 2C) demonstrated that longer time and elevated L/S ratio are necessary to extract a high amount of TP from PP.

The use of the quadratic model to describe the experimental data allowed the definition of three optimized conditions for the maximization of the extraction of TP from PP. These are an ethanol proportion of 80%, an extraction time of 150 min, and a ratio of 30 mL/g.

According to Amado et al. [43], the optimal TPC from PP was achieved at 34 min using an ethanol concentration of 71.2%. Paleologou et al. [9] described water/ethanol mixtures of 59% and an L/S ratio of 84 mL/g to extract the maximum TPC from PP. Frontuto et al. [4] found a lower optimal concentration of ethanol (52–54%), but a long time of 233 min, using the solid–liquid method to extract phenolics from PP. Apart from the parameters studied, different factors are involved in the extraction of phenolics from PP such as the origin, variety, and growing conditions of the potatoes.

### 3.3. Total Phenolic and Total Flavonoid Contents and Antioxidant Activity of Optimized PP Extract

The TPC of the optimized PP extract was estimated at 204.41 ± 86.54 mg GAE/100 g DW, while the TFC was 21.47 ± 0.76 mg QE/100 g DW. These results agree with those previously mentioned in literature. Indeed, Friedman et al. [44] showed that TPC ranged from 11 to 2840 mg of GAE/100 g of powder weight (organic peels) and from 1130 to 3440 mg GAE/100 g of powder weight (conventional peels). According to Bădărău et al. [45], the highest value of TPC was 1079 mg GAE/100 g DW. Similarly, the best TPC was reported in the acidified ethanolic extract (~1400 mg GAE/100 g) [46]. In 2019, Samotyja [47] found a content of 4050 mg GAE /100 g DW of the extract and Venturi et al. [48] a content of 400 mg GAE/100 g DW. Joly et al. [49] revealed that a considerable TPC value was found with the heat processing of methanol extract (383 mg chlorogenic acid equivalents/100 g of sample powder). In the same year, the amount recorded by He et al. [50] was 300 mg GAE/100 g of alcohol-insoluble residues. Several researchers have revealed that colored potato varieties are richer in TPC [10,51]. In this line, genotypes with red or purple flesh and skin had three to four times higher TPC than non-pigmented tubers [51]. Furthermore, the TPC in PP extracts using solid–liquid extraction increased from 328 mg GAE/100 g DW to 767 mg GAE/100 g DW in the LadyRosetta variety, whereas for the cream-skinned LadyClaire variety, the TPC increased from 217 mg GAE/100 g DW to 424 mg GAE/100 g DW [10].

The fluctuation in TPC, as in the case of the other components, from PP is eventually assigned to diverse factors, namely, peeling techniques, agronomic and other environmental factors, and the varietal differences [9]. The extraction process and its conditions also influence the levels of TPC in PP. Wang et al. [3] showed that the TPC in PP was about 48% higher using direct ultrasound-assisted extraction (930 mg GAE/100 g DW) compared to conventional shaking extraction (626 mg GAE/100 g DW).

Regarding the kind of phenolic compounds in PP, they comprise several phenolic compounds in free and bound forms. The major phenolic acids identified were chlorogenic, gallic, protocatechuic, and caffeic acids [52].

In the case of flavonoids, and according to Choi et al. [53], the highest TFC in PP was 602 mg of QE/100 g of sample, and according to Silva-Beltrán et al. [46] the content obtained with acidified ethanolic extract was 300 mg of QE/100 g. Moreover, the TFC varied from 780 to 2300 mg of QE/100 g of powder weight (conventional peels) and from 870 to 2970 mg of QE/100 g of powder weight (organic peels) [44].

PP polyphenolic extracts exert their antioxidant capacity in a dose-dependent way, with a direct correlation between the antioxidant capacity and the phenolic content [54]. The currently studied PP extract, rich in phenolics, presented a substantial antioxidant power in the tested assays, with the low IC_50_ value of 10.65 ± 0.21 µg/mL in the phosphomolybdate assay, while in the DPPH scavenging assay, the IC_50_ value was 179.75 ± 3.18 µg/mL.

Antioxidants can be affected by many factors such as enzymes, pH, and temperature [50]. Javed et al. [52] reported that temperature decreased the antioxidant capacity of potato peel extracts. Kumari et al. [10] demonstrated that the DPPH^•^ free radical activity was affected by the potato variety and the extraction method used. Extracts of two PP varieties obtained by ultrasound-assisted extraction (UAE) almost doubled the DPPH^•^ radical scavenging activity compared to solid–liquid extraction for LadyRosetta and LadyClaire potato peel varieties. Moreover, the (UAE) LadyRosetta variety extract presented major antioxidant activity using DPPH assay (3.51 ± 0.00 mg TE/g DW) than the extract from the LadyClaire variety (1.75 ± 0.05 mg TE/g DW). According to Frontuto et al. [4], the values of the factors that maximize the DPPH activity were 62% ethanol, 240 min, and 50 °C for S/L ratio extraction (803.08 mg AAE/kg FWPP), and 57% ethanol, 240 min, and 50 °C using pulsed electric fields-assisted extraction (877.17/kg FWPP). de Andrade Lima et al. [55] found that the DPPH radical scavenging activity of the supercritical fluid extracts depends on the extraction conditions such as the extracting solvent. They showed that the application of pulsed electric fields extraction pre-treatment to potato peels markedly enhanced the antioxidant activity (9%) of the extracts.

Similarly, the antiradical activity (AAR) was significantly higher in the water/ethanol extract (56.4 ± 1.30 μmol DPPH/g DW) compared with the water/glycerol extract (43.89 ± 0.00 μmol DPPH/g DW) [9]. In addition, Lakka et al. [11] found that the AAR of PP was 16.37 ± 0.49 µmol DPPH g/DW using hydroxypropyl β-cyclodextrin (HP-β-CD), 25.26 ± 0.76 µmol DPPH g/DW using ethanol 60%, and 23.04 ± 0.69 µmol DPPH g/DW using methanol 60%.

Sampaio et al. [54] reported that bounded phenolics showed an efficient antioxidant capacity equal to or better than the free ones. The potato peels of colored varieties often showed higher antioxidant capacities because of their high anthocyanin content [56]. Based on its phytochemical contents, the PP can have potential as functional food ingredients or food health, especially thanks to their activity as antioxidants.

### 3.4. Fortification of Yogurts with PP

In order to check the cost-effectiveness of the exploitation of PP as a natural ingredient for yogurt fortification, an analysis of the fortified yogurt was carried out, namely, moisture, ash, total solids contents, pH, titratable acidity, phenolic contents, antioxidant capacity, and sensorial analysis.

#### 3.4.1. Composition and Physicochemical Analysis

The results of the composition and physicochemical parameters for the assessed yogurt samples are presented in Table 4.

The moisture value recorded in the yogurt sample enriched by PP (82%) was close to the ones mentioned by Jung et al. [28] (almost 84%) for yogurt fortified with red ginseng extract, and to the content stated by Bhat et al. [29] (almost 80%) when studying yogurt fortified with psyllium (*Plantago ovate*) husk.

Ash amount is an evaluation of the total mineral content in yogurt. It is important because the mineral content of yogurt impacts its physiochemical parameters [29]. The fortification of the yogurt with the PP powder resulted in an increase in ash content (0.64%), as noted from the results compared to the control (0.55%) (Table 4). This may be attributed to the high mineral content of PP. Martinez-Fernandez et al. [57] recorded a content of 4.3 wt % of ash in PP. These findings are in accordance with those reported by Jung et al. [28], who found an ash content of 0.87% in the control and 0.95% in the yogurt enriched with 2% red ginseng extract. Similarly, Bhat et al. [29] found that the fortified yogurt by *Psyllium husk* at 0.1% was richer in ash (0.84%) comparatively to the control (0.77%).

Total solids are evidence of the dry matter of yogurt samples. The results showed that the PP powder increased the total soluble solids from 15% to 17%. Jung et al. [28] reported 14.45% of total solids for the control and 15.57% for the yogurt supplemented with 2% of red ginseng extract. The total solids content in the yogurts studied by Bhat et al. [29] was 13.09% and 13.35% for the control and fortified yogurt with *Psyllium husk* at 0.7%, respectively. Moreover, the yogurt samples with apple peel polyphenol extract had a slightly increase of total solids content than the control [25].

pH and titratable acidity were also measured in the yogurts (Table 4). The pH value was higher in the control yogurt (4.54) that in the PP-added yogurt (4.30). The acidity of dairy products must be between 0.6% and 1.5%, and consumers opt for fermented products with pH varying between 4.2 and 4.4 [21]. Even so, the PP yogurt showed lower values for these parameters. This decrease in pH may be assigned to the presence of fermentable compounds in PP that were metabolized by the lactic acid bacteria, leading to organic acid formation [13]. In addition, increasing the pH and reducing the acidity by adding PP can be attributed to their positive effect on the activity of starter bacteria in yogurt [24]. The same trend was observed in a previous study reporting that the pH value diminished in the yogurts containing bioactive aqueous extract of *Pleurotus ostreatus* [30]. Similar findings were given by Barkallah et al. [15] when investigating the incorporation of Spirulina into yogurts, and this was attributed to the different buffering capacity effects of the treatments. In addition, the control had a high pH value of 4.56 compared to yogurts prepared with apple peel polyphenol extract at different concentrations (4.51–4.36) [25]. In 2021, Ahmed et al. [13] revealed that fortified yogurts with argel (*Solenostemma argel* Hayne) leaf extract had a lower pH than the control. The cryo-concentrated strawberry pulp addition in yogurt also promoted a decrease in the pH, and hence, an increase in the acidity [21]. Thus, the strain of lactic acid bacteria in the yogurt, the duration, the temperature of incubation, and the ingredients incorporated into the yogurt influenced its pH [17].

The highest value of titratable acidity was recorded in the yogurt fortified by PP powder (0.85%) compared to the control (0.81%). The fortification of apple peel polyphenol extract slightly increased the acidity percentage of the yogurt (0.79–1.01%) compared to the control sample (0.82%) [25]. The yogurt enriched by argel also showed higher acidity than the control [13]. More energy can be used for the growth of the lactic acid bacteria population when there are more sugar components and more simple molecules extracted. [17].

High total solids content leads to increased acid production; moreover, PP contains soluble fibers [58], which could be easily degraded by the bacteria, resulting in the production of organic acids [29] including lactic acid, leading to an increase intitratable acidity and a decrease in pH. This was also enhanced by different secondary metabolites such as polyphenols and organic acids [13], which are presented in PP.

#### 3.4.2. Phenolic Content and Antioxidant Activity

The fortification of yogurt with bioactive substances abundant in plants like PP may improve its health interests.

The results showed that yogurt formulated with PP had significantly (*p* < 0.05) increased TPC (10.30 ± 0.06 mg GAE/100 g DW) compared to the control yogurt (5.66 ± 0.23 mg GAE/100 g DW). Similarly, the yogurt supplemented with grape (*Vitis labrusca* L.) pomace demonstrated significantly higher TPC values (52.04 mg GAE/100 g) than the control yogurt (11.01 mg GAE/100 g) [26]. A TPC value of 3% apple pomace fortified yogurt was higher (0.75 mg GAE/100 g) than that of the control one (0.041 mg GAE/100 g) [31].

The enrichment of yogurt with grape and aronia juice enhanced TPC from 30% to 33% and up to 49% when blueberry juice was used. Furthermore, the control showed lower TPC (0.043 mg GAE/100 g) levels compared to the yogurt with grape juice (0.056 mg GAE/100 g), yogurt with aronia juice (0.057 mg GAE/100 g), and yogurt with blueberry juice (0.065 ± mg GAE/100 g) [27]. Likewise, Ahmed et al. [13] confirmed that enriching yogurts with natural products increased their TPC, being 33.2% higher in yogurt added with 0.2 g/100 mL argel leaf extract compared to the control.

Flavonoids are found substantially in vegetable foods and are very strong free radical scavengers. The TFC in the yogurt fortified with PP (3.69 ± 0.03 mg EQ/100 g DW) was significantly higher (*p* < 0.05) than that of the control (1.29 ± 0.02 mg EQ/100 g DW). Similar findings were formerly stated by Barakat and Hassan [18], where the incorporation of pumpkin pulp into yogurt resulted in higher TP and TF contents.

Oxidative reactions can negatively impact the flavor, bioactive compounds, and nutrients, and they can also produce potentially toxic oxidation products [25]. Hence, it is interesting to search for antioxidant sources such as PP with notable TPC and TFC that can develop antioxidant capacity in the food matrix while avoiding the oxidative reactions. The results of the antioxidant capacity assessed by the total antioxidant activity and DPPH radical scavenging activity of the yogurts are given in Table 3. The addition of the PP powder, rich in antioxidant compounds, statistically significantly (*p* < 0.05) enhanced the antioxidant power of the fortified yogurt, which was monitored by both antioxidant assays, phosphomolybdate assay (IC_50_ = 29.58 ± 0.65 mg/ mL) and DPPH assay (IC_50_ = 15.86 ± 0.04 mg /mL). The higher antioxidant activity in the PP yogurt could stem from the phytochemical content of PP and the microbial metabolic activity that releases some bound bioactive molecules. The metabolism of phenolics by the yogurt cultures may involve the hydrolysis of flavonoid glycosides or C-ring cleavage and the liberation of simple phenolics, including phenolic acids favoring the enhancement of antioxidant capacity [23]. For this reason, several vegetable extracts rich in phenolics have been included in dairy products to improve their antioxidant capacity.

The considerable quantities of phenolics are the main reason for the high amounts of antioxidant capacity, as there is a direct correlation between the content of phenolics and the antioxidant effect. Hence, the antioxidant capacity of the purslane extract was significantly augmented from 0.5% to 2%, due to the high TPC in the purslane than in the control [24].

Caleja et al. [14] discovered that yogurts containing fennel and chamomile decoctions awarded better antioxidant properties than yogurts with a synthetic additive (E202). These authors noted a reduced value of antioxidant power (IC_50_ = 94 ± 4 mg/mL for fennel decoction and 45 ± 3 mg/mL for chamomile decoction) compared to the control (IC_50_ = 195 ± 5 mg/mL). Similarly, the radical scavenging capacity of yogurt enriched with red ginseng extract (89.44 to 94.80%) was higher than that of the control (63.63%) [28]. Barkallah et al. [15] found that the yogurt supplemented with 0.25% of *Spirulina* produced lower IC_50_ (30 mg/mL) than the control with IC_50_ > 50 mg/mL. Furthermore, the sample incorporating grape (*Vitis labrusca* L.) pomace at 5% (262.69 mg/mL) improved the anti-DPPH capacity of the yogurt [26]. In the study conducted by Dimitrellou et al. [27], the control yogurt presented the lowest DPPH scavenging activity (14.7 ± 0.9 TE/100 g), while yogurt with blueberry juice presented the highest one (33.0 ± 0.2 TE/100 g), followed by yogurt with aronia juice (30.0 ± 0.8 TE/100 g) and yogurt with grape juice (21.5 ± 1.8 TE/100 g). Likewise, Ahmed et al. [13] noticed that the DPPH^•^ radical scavenging activity was higher by 108.6% in yogurt containing 0.2% argel leaf extract compared to the control. The enrichment with hazelnut skin also enhanced the TPC and antioxidant capacity of yogurts [19]. The supplementation of yogurt with cryo-concentrated strawberry pulp resulted in a product with 3-fold more anthocyanins and antioxidant capacity [21]. It is important to emphasize that the uptake of rice berry yogurt induced an acute rise in plasma antioxidant capacity in healthy adults [16].

The fluctuation in the antioxidant capacity of yogurts enriched with various plant matrices may be due to numerous factors such as their origin and composition. Furthermore, milk protein proteolysis and organic acid formation derived from microbial metabolic action during fermentation and cold storage may have contributed to the increased antioxidant capacity [19].

To the authors’ knowledge, there are no studies dealing with the enrichment of dairy products with PP powder or their extracts. However, the latter were used as a natural antioxidant to prevent oxidation in other food products. Franco et al. [59] revealed that the incorporation of ethanolic extracts of potato peels to soybean oil reduced lipid oxidation and showed higher antioxidant activity than butylated hydroxytoluene (BHT), results that were supported by Samotyja [47] and Amado et al. [43]. Moreover, potato peel flour (0–2%) was used in the fortification of beef patties to enhance the antioxidant properties [60]. Starch-based active films made with phenolic extracts from PP increase the antioxidant capacity of these films used to pack smoked fish fillets by upto 48% [61]. Hence, the supplementation of yogurt with PP may increase its functionality by increasing its phenolic content concomitantly with the elevation of antioxidant activity.

#### 3.4.3. Sensorial Evaluation

The expert panel conducted two different analyses: (i) the sensory analysis of the formulated yogurts in order to describe their characteristics (color, smell, aroma, sweetness, acidity, texture, and consistency), and to evaluate their intensities; and (ii) an acceptability study, which is the preference ranking of the three yogurts (overall quality).

**Figure 3:**
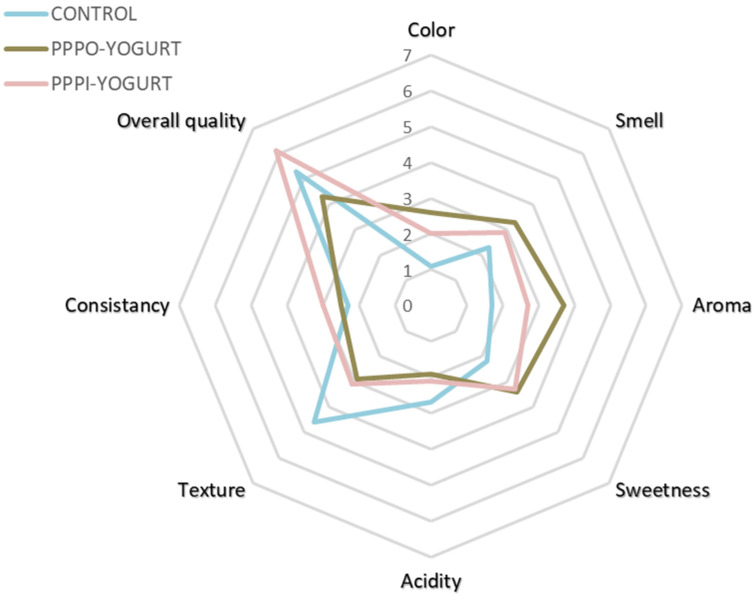
Spider graphs of the sensory profile of the formulated yogurt samples. PPP-YOGURT: yogurt fortified with potato peel powder, PPI-YOGURT: yogurt fortified with potato peel pieces.

The sensorial characteristics of the yogurt fortified with PP (powder and pieces) and the control are displayed in Figure 3. The PP affected the sensorial features of the yogurts and enhanced the color, smell, aroma, sweetness, consistency, and overall quality of the formulated yogurts compared to the control.

**Figure 4:**
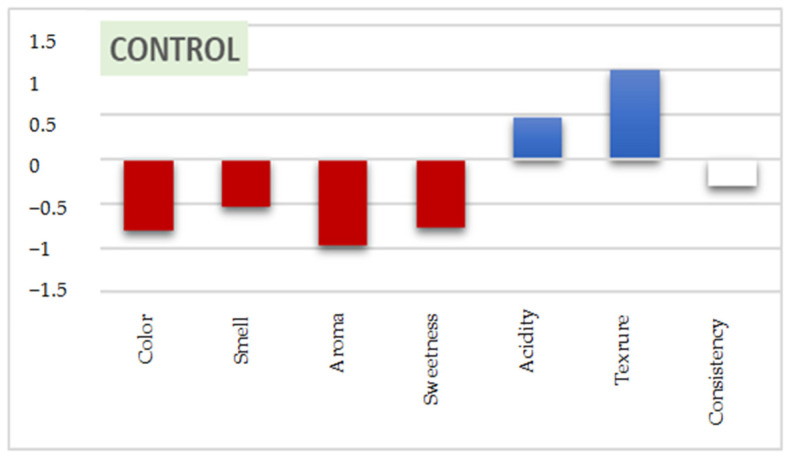

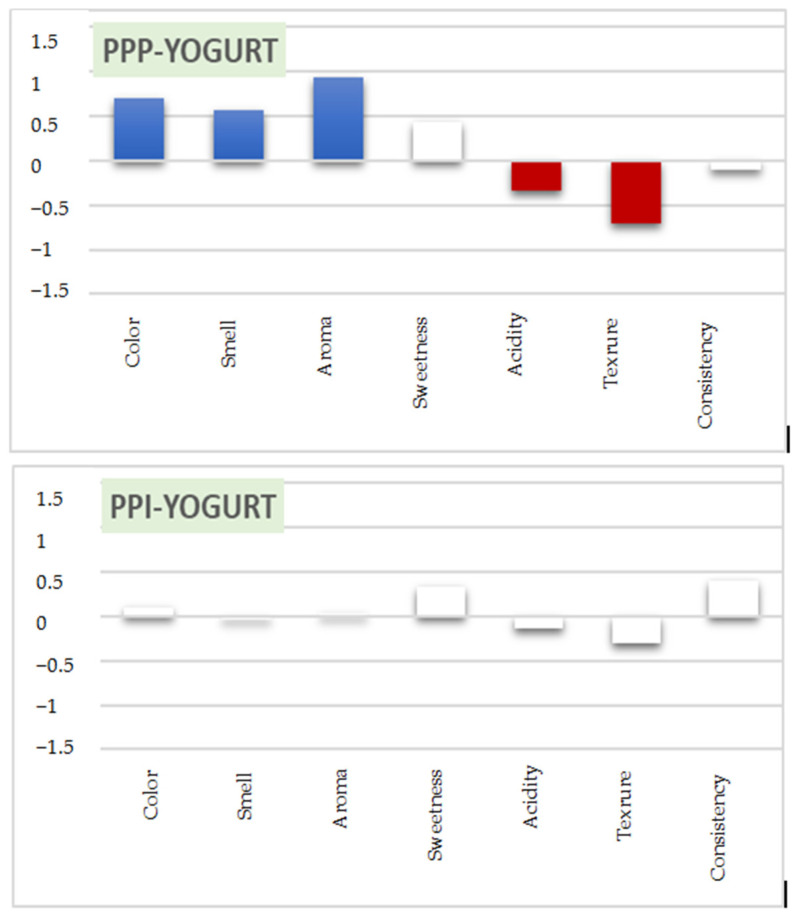
Results of the yogurt characterization. PPP-YOGURT: yogurt fortified with potato peel powder, PPI-YOGURT: yogurt fortified with potato peel pieces. The low intensities of the evaluated characteristics are designated in red, those of high intensity in blue, and those of medium intensity in white.

The results of the product characterization revealed that the control is characterized by color, smell, aroma, and sweetness of low intensity compared to the other two samples. As for the yogurt enriched with the PP pieces, all sensory characteristics are of medium intensity. The yogurt enriched with PP powder has high color, smell, and aroma; its acidity and texture are low; and its sweetness and consistency are of medium intensity (Figure 4).

In accordance with other findings, an improvement in the sensorial properties was mentioned in yogurt fortified with apple peel and grape seed powder [32], apple peel polyphenol extract [25], and argel (*Solenostemma argel* Hayne) leaf extract [13].

**Figure 5:**
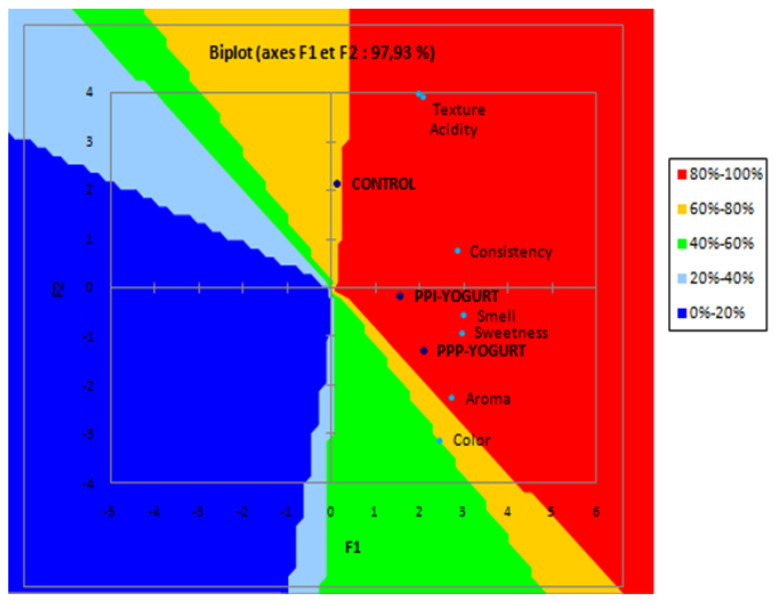
Preference mapping of the three assessed yogurts. PPP-YOGURT: yogurt fortified with potato peel powder, PPI-YOGURT: yogurt fortified with potato peel pieces. The non-enriched control yogurt is located in the yellow zone corresponding to the judges’ satisfaction percentages ranging from 60% to 80%. Enriched yogurts are in the red zone, which corresponds to the percentages of satisfied judges ranging from 80% to 100%. For characteristics, those that correlate are close, such as texture and acidity; smell and sweetness; aroma and color.

The External Preference Mapping (PREFMAP) method allows relating the preferences expressed by the consumer to sensory characteristics. This approach is crucial as researchers rely on it to adapt products to the tastes of the consumer, and it also makes it possible to know the sensory characteristics that have an influence on the acceptability of the product (positive or negative influence). For this purpose, this method was created (Figure 5) after applying a principal component analysis (PCA) and grouping the panelists according to their preferences by performing agglomerative hierarchical clustering (AHC).

According to the results obtained, the percentage of satisfaction of the panelists was 100% for the yogurt enriched with potato peel pieces (PPI-YOGURT), it was the most appreciated since it is characterized by its smell, sweetness, and consistency of high intensity compared to the other yogurts. As for the yogurt with potato peel powder (PPP-YOGURT), the percentage of satisfied judges was 80% due to the high intensity of its color and aroma. However, only 60% of the judges appreciated the unenriched yogurt (control). This yogurt was the least preferred because of its high acidity and smooth texture compared to the other products. These results are consistent with those of the product characterization.

Flavored yogurt, usually with fruit pieces, is more widely consumed by the Algerian population compared to plain yogurt. These factors may help explain the panelists’ preference for the PP-enriched yogurts. The addition of natural agents such as PP powder or pieces may improve the nutritional, bioactive, and sensorial profile of the yogurt.

It should be noted that yogurt fortification with PP powder resulted in an increase in overall acceptability in terms of, in particular, consistency and sweetness. This can be attributed to the composition of the PP, which showed high levels of dietary fiber and proteins [54]. Jeddou et al. [62] developed PP-enriched cakes in which water binding, fat absorption properties, and the overall acceptability were improved. Meanwhile, some researchers have highlighted the positive impact of fiber enrichment on the body and consistency of yogurt [29,63]. Several researchers have also developed yogurts fortified with different natural byproducts and observed that their addition had a positive impact on the appearance, taste, and overall acceptability of the yogurt samples [25,26,32].

## 4. Conclusions

Economically discarded potato peel (PP) waste produced in Algeria was investigated, on the one hand, for the optimization of the main phenolic compound extraction parameters, and on the other hand, to fortify yogurt with PP as a natural ingredient to improve its physical, nutritional, antioxidant, and sensorial characteristics. The best conditions for the extraction of phenolic compounds from PP are ethanol at 80% (*v/v*) for 150 min with a ratio of 1 g/30 mL. The extract obtained had a considerable scavenger effect against free radicals. In fact, when PP was included in the yogurt formulation, the health potential of the yogurt was improved by increasing its TPC, TFC, and antioxidant capacity.

Otherwise, the yogurt with PP differed from the control in terms of its physicochemical properties. The enrichment of yogurts with PP resulted in an increase in total solids, ash content, and titratable acidity, and the pH value decreased.

The results obtained affirmed the cost-effectiveness of the application of PP as a natural food fortifier. Suitable for food uses, the PP powder has not altered the sensorial parameters of the assessed dairy product; on the contrary, it has improved some evaluated attributes such as consistency and overall quality, which is auspicious in terms of consumer acceptability and can be taken into consideration by the food industry. Therefore, PP, which is a food waste, can be used as a byproduct with good results to formulate functional yogurt with improved physicochemical properties, and to enhance its phytochemical profile and increase its antioxidant potential and sensory qualities.

Future experiments on the optimal quantities to be used in yogurt recipes and other analyses to determine the content of other compounds such as fiber, carbohydrates, proteins, and minerals are needed. The assessment of the variation of the different parameters during the storage period is another substantial parameter to be evaluated.

## Figures and Tables

**Table 1:** Series adopted for the factors in the preliminary study, coded, and real values for central composite design (CCD) of PP extraction.

Factors	Adopted Series					
Ethanol concentration, %, *v/v* (*E*)	20	40	60	80	100	
Extraction time, min (*T*)	30	60	90	120	150	180
Liquid/solid ratio, mL/g (*R*)	10	20	30	40	50	60
Factors	Coded levels					
	−1		0		+1	
Ethanol concentration, %, *v/v* (*E*)	20		50		80	
Extraction time, min (*T*)	90		120		150	
Liquid/solid ratio, mL/g *(R)*	10		20		30	

1 (low), 0 (center point), and +1 (high), were the codes of factor levels.

**Table 2:** Experimental and predicted parameters of TPC (mg/100 g DW) extraction yields from PP using central composite design.

Configuration	Ethanol (C) (%)	Time (min)	L/S Ratio (mL/g)	Experimental Values	Predicted Values
	*E*	*T*	*R*		
0a0	50	90	20	36.50	26.82
00A	50	120	30	102.29	98.06
++−	80	150	10	133.04	134.82
000	50	120	20	50.60	41.38
000	50	120	20	39.64	41.38
000	50	120	20	45.94	41.38
−−+	20	90	30	41.15	39.70
−−−	20	90	10	49.46	59.66
+−−	80	90	10	46.63	39.49
+++	80	150	30	217.56	207.69
A00	80	120	20	40.57	47.73
000	50	120	20	32.27	41.38
+−+	80	90	30	71.64	79.71
−++	20	150	30	92.15	99.62
000	50	120	20	42.36	41.38
0A0	50	150	20	96.09	104.44
000	50	120	20	44.85	41.38
a00	20	120	20	12.27	03.78
00a	50	120	10	68.70	71.60
−+−	20	150	10	94.65	86.91

**Table 3:** Analysis of the variance of the experimental results of PP extract.

Source	Degree of Freedom	Sum of Squares	*F*-Value	*p*-Value Prob > F
Model	9	39,361.438	46.3129	<0.0001
Ethanol (C) (%) (40,80) (*E*)	1	4829.446	51.14	<0.0001
Time (min) (60,120) (*T*)	1	15,062.973	159.5082	<0.0001
S/L ratio (g/mL) (20,40) (*R*)	1	1750.594	18.5378	0.0015
Ethanol (C) (%) × Time (min) (*E*× *T*)	1	2316.762	24.5332	0.0006
Ethanol (C) (%) × S/L ratio (g/mL) (*E* × *R*)	1	1810.214	19.1692	0.0014
Time(min) × S/L ratio (g/mL) (*T* × *R*)	1	533.338	5.6478	0.0388
Ethanol(C)(%) × Ethanol(C) (%) (*E* × *E*)	1	711.299	7.5323	0.0207
Time (min) × Time (min) (*T* × *T*)	1	1556.699	16.4846	0.0023
S/L ratio (g/mL) × S/L ratio (g/mL)(*R* × *R*)	1	5082.923	53.8253	<0.0001
Residual	10	944.336		
Lack of fit	5	748.59		0.0836
Pure error	5	195.74	39.149	
Total error	10	944.34		
Total	19	40,305.775		
R^2^	0.9765			
Adjusted R^2^	0.9554			

**Table 4:** Composition, physicochemical parameters, total phenolic content (TPC), total flavonoid content (TFC), and IC_50_ (µg/mL) values found in the DPPH and phosphomolybdate assays for the yogurts fortified with PP.

	PPYogurt	Control
**Composition**		
Moisture (%)	82 ± 0.92 ^b^	89 ± 1.13 ^a^
Ash (%)	0.64 ± 0.02 ^a^	0.55 ± 0.01 ^b^
Total solids (%)	17 ± 0.17 ^a^	15 ± 0.04 ^b^
**Physicochemical parameters**		
pH	4.30 ± 0.01 ^a^	4.54 ± 0.01 ^a^
Titratable acidity (%)	0.85 ± 0.03 ^a^	0.81 ± 0.02 ^a^
**Phenolic content**		
Total phenolics (mg GAE/100 g DW)	10.30 ± 0.06 ^a^	5.66 ± 0.23 ^b^
Total flavonoids (mg QE/100 g DW)	3.69 ± 0.03 ^a^	1.29 ± 0.02 ^b^
**Antioxidant activity**		
Total antioxidant activity IC_50_ (mg/mL)	29.58 ± 0.65 ^a^	318.65 ± 5.75 ^b^
Radical scavenging capacityIC_50_ (mg/mL)	15.86 ± 0.04 ^a^	82.06 ± 0.71 ^b^

The mean of values in the same line assigned with different letters indicate significant differences (*p* < 0.05).

## Data Availability

Data is contained within the article.
